# A Systems Thinking approach for the creation of effective competency-based medical education programs

**DOI:** 10.11604/pamj.2022.41.203.28896

**Published:** 2022-03-14

**Authors:** Imad Salah Hassan, Amna Khairy AbdulKareem, Noon Hatim khalid Alrabee, Sahar Faisal Mansour, Safaa Abdelmoneim Fadlelmoula, Eman Abdel-Azim Elhassan, Mukhtar Mohamed Abdelgadir, Mohamed Abdelfatah Mohamed, Sarah Ali Adam, Fatima Obied Bedawi, Mohammed-Elfatih Ahmed Yousif, Roa Awadalla Ahmed, Taysir Altaib Kashif

**Affiliations:** 1 King Abdulaziz Medical City, Riyadh, Saudi Arabia,; 2 Al Tababa Competency-based Training Unit, Khartoum, Sudan

**Keywords:** Competency-based medical education, Systems Thinking, biomatrix tool

## Abstract

Successful and sustainable implementation of Competency-based Medical Education (CBME) programs is a significant and daunting challenge facing medical education worldwide. Our manuscript endorses for the first time, Systems Thinking as a concept for transforming and redesigning CBME programs employing the full 7-system elements as advocated by the Biomatrix Systems Theory. The majority of internationally recommended actions and processes for such an endeavor are highlighted, each within its system element. New innovative ideas such as having competency-structured clinical training activities as well as re-writing medical textbooks following a novel competency-based roadmap for their disease monographs etc. are also highlighted. Furthermore, the need for innovative partnerships as well as novel medical rotations that may facilitate the creation of “master clinicians” are also stressed.

## Perspectives

**The CanMEDS as a competency-based, outcome-focused training paradigm:** competency-based, outcome-focused training or Competency-based Medical Education (CBME) sprung to the headlines of the healthcare agenda as the preferred tool to build and mold the clinicians of the future. It signaled a paradigm shift in the concept of building training curricula with the curriculum “built-in-reverse”-starting with the desired outcome first. Simplistically, competency may be defined as a standardized requirement for an individual to properly perform a specific job. It encompasses a combination of knowledge, skills, and attitude (behavior) that are indispensable for the successful accomplishment of a required task or duty [1]. The CanMEDS Framework [2] (the Canadian Medical Education Directives for Specialists) is one of at least 10 competency-based, outcome-focused frameworks for training. Others include that of the United States (ACGME), England (tomorrow's doctors), the Gulf States (the gulf cooperative council curriculum), etc. Unlike the others, the CanMEDS gained the most popularity world-wide. It is defined as an “educational framework that describes the abilities physicians require to effectively meet the health care needs of the people they serve” [2]. The resulting framework consists of competencies organized under seven major roles of a clinician. They are medical expert (the central role), communicator, collaborator, leader (previously manager), health advocate, scholar, and professional. Under each competency, multiple sub-competencies emerge resulting in more than 50 essential skills to be learned and mastered. It is envisaged that the need for these competencies is so dynamic that a single patient-encounter will entail proficiently utilizing all these skills if at-all a successful and happy-conclusion-the desired outcome- is to materialize: “Doing the right thing, at the right time, in the right way, in complex situations” [2].

**Challenges in designing and running CBME programs:** establishing effective and sustainable CBME programs has been a challenge worldwide. A CBME program is envisaged to be not only outcome-focused but is learner-centered with a clear de-emphasizes on time-based training [1]. Highlighted barriers are numerous and include non-supportive regulatory policies, administrative and financial difficulties, variations in CBME frameworks and definitions, acceptance by staff and trainees, unavailability of competent faculty for teaching and coaching, issues with adequate, agreed-upon, and objective assessment tools of “in-vitro” and “in-vivo” outcomes, etc. [3,4]. The latter is of major significance as the assessment is a cardinal element in facilitating and encouraging both trainee and faculty learning and empowerment [5]. Proposed solutions to facilitate the incorporation and implementation of a CBME program are abound with multiple expert commentaries and recommendations [6-10]. Several recommendations point to a Systems Thinking approach though mostly implicitly and without articulating all the System elements of importance.

### What is Systems Thinking?

Systems Thinking is a paradigm and revolutionary tool for “creativity and learning” and for “solving/dissolving” problems in complex social systems. It is considered a foundational requirement for transformational leadership and for maximizing system effectiveness [11]. It first appeared in the business and management arena but has spread to all disciplines or “systems” especially when the human factor is a pivotal element e.g. in social systems. It was highly-publicized by a nobel prize winner, professor Peter Senge in his book, the fifth discipline [12]. A critical component underscoring the success of the Systems Thinking approach is its focus on staff (the human factor) rather than the service-the so-called System Intelligence coupled with a great emphasis on a multidisciplinary mode of action. A system is simply defined as an entity with interrelated and interdependent parts that are working together to achieve a common purpose. Any change in one part of the system e.g. creation of a highly-competent workforce affects the part and the whole system. In a more refined depiction, Arnold and Wade defined Systems Thinking as “a system of synergistic analytic skills used to improve the capability of identifying and understanding systems, predicting their behaviors, and devising modifications to them in order to produce the desired effects” [13]. Healthcare is considered a system with numerous structures and processes that are integrated to achieve wellness as an ultimate purpose. Systems Thinking approaches are the advocated tools for all healthcare transformative actions [14,15] including medical education [15]. Systems Thinking is a holistic approach to better understand the “big-picture” and how the system elements interact with each other over time, the root-causes of system defects, and the right approach for a highly-effective problem-solving intervention and “system redesign”. Intriguingly, and like Competency-Based Medical Education, Systems Thinking is all about outcomes. Its tools for “system redesign” enable its users to radically create the results and outcomes they truly desire thanks to a methodology for selecting and focusing on the right “high-leverage areas”. A leverage point is considered “high” if a “quantitatively equivalent input” at another system area results in a (comparatively) significantly inferior impact. One of the tools for identifying and selecting high-leverage areas is called the biomatrix tool, a tool stemming from the science of the Biomatrix Systems Theory [16]. Its seven components constitute the building blocks for any effective system be it a clinical unit, education program, hospital, organization, government, etc. Unlike the classic description of a system with only its 3 basic components: the structure, process and outcome, the biomatrix tool elegantly incorporates 4 extra indispensable elements for a comprehensive and successful system design or redesign. One may thus utilize it to build a successful and sustainable competency-based training program.

**Building a competency-based training program using Systems Thinking:** most of the literature on Systems Thinking and CBME focuses primarily on using the concept in formulating teaching, training, and assessment activities (processes of work) [17-19]. Furthermore, limited success with CBME programs was clearly related to a narrow focus on the processes of implementation rather than a holistic endeavor to improve the system [20-22]. The well-known quote that “every system is perfectly designed to get the results it gets” holds very true in CBME programs. A Systems Thinking approach is necessary for transformative action in medical education [15]. Table 1 and Table 1 (suite) depicts the seven system elements and their relevant practical administrative components and actions for a competency-based program. Having a clear aim coupled with a robust organizational culture is paramount [23]. The “organizational culture” impact on realizing the desired patient, institutional or regulatory outcomes is supported by systematic reviews [24]. Old structures (system anatomy or organogram) maintain “the unacceptable status quo”! Thus, changing system structures is an essential step if new aims or processes are to be incorporated. Lack of competent faculty remains a major limiting “structural” factor in many CBME programs [25,26]. New processes invite another important concept in system improvement namely Design Thinking. Like CBME and Systems Thinking, Design Thinking is also an outcome-focused approach. Its place in medical education is squarely hinged on its focus on four important aspects of system redesign namely: 1) empathy entailing a deep understanding of the needs of the end-users thus resonating with both learner- and teacher-centeredness (needs assessment); 2) producing creative solutions through collective brainstorming; 3) prototyping or experimentation and finally; 4) actively acquiring feedback from the target users and timely adjustments [27]. Innovative redesigning of teaching and training practices and even reading materials of trainees and faculty are vital. Thus, structuring ward rounds, morning meetings, case presentations and management, etc. using competency-headings facilitate concept-comprehension and may enhance its incorporation in the trainee´s cognition and practice [28]. Table 2 depicts a competency-structured presentation or discussion for the topic of bronchial asthma. Likewise, using competency-headings in writing textbooks´ disease monographs instead of the old-styled headings of, for example, definitions, epidemiology, pathophysiology, clinical presentation, treatment, etc. may serve a similar purpose. Dedicated competency-based workshops and new or redesigned rotations, etc. are also vital [29,30].

**Table 1:** CanMEDS competency-based training program biomatrix elements and administrative actions

Biomatrix item	Description	Component or action
**Aims (vision)**	**The outcome(s):** the results that the system wants to achieve. Aims create focus	Healthcare transformation
**Ethos**	**Organizational culture:** its unique expectations, and values and is expressed in its self-image- “as you think, so you will become”	Master clinicians
**Structure**	**The organogram:** the anatomy of a system	Legislative policies and regulations
		CanMEDS training committee
		CanMEDS trainee curriculum
		CanMEDS faculty training program
		CanMEDS structured teaching and training ebooks
		Educationalists/educational consultants
		Simulation laboratory
		Mentorship and coaching experts
		IT platforms
		Monitoring and assessment unit
**Process**	**The activities:** describes the activities of the system-the activities involved in the delivery of services (training) to the customers.	CanMEDS competencies induction program for faculty and first-year residents
		CanMEDS teachable moments
		Competency presentations and workshops e.g. in the communicator role, professionalism etc.
		CanMEDS-structured case discussion in the morning meeting
		CanMEDS-structured clinical topic presentation during the half-days
		CanMEDS structured ward round
		Scholar role activities: evidence-based literature searching workshops in a computer laboratory, teaching skills workshop, research skills workshop etc.
		Professional role activities: ethical practice/case vignettes, self-care etc.
		Leadership role activities: leadership skills, service improvement skills (quality and safety improvement tools like audit), career management, committee membership etc.
		Communication role activities: written (history and physical, follow-up notes, discharge summaries etc.), patient-centered communication, hand-over, therapeutic communication, motivational communication, breaking-bad news, disclosure of error, dealing with angry patients or relatives etc.
		New non-clinical rotations: EBM rotation, research and audit, medical technology, medical bioethics, community health, medical education etc.

**Table 1(suite):** CanMEDS competency-based training program biomatrix elements and administrative actions

Biomatrix item	Description	Component or action
**Resources**	**Material and intellectual assets:** refer to the resources of the organization, such as its capital equipment, financial resources, intellectual property, staff capabilities etc.	Financial resources
		CanMEDS skilled faculty
		Educationalist/educational consultants
		CanMEDS audiovisual resources
		CanMEDS case vignettes
		CanMEDS website, blogs etc.
**Environment**	**Local and surrounding facilitators and barriers:** (the latter need to be resolved at the outset)	Incentive and championship program for faculty and trainees.
		Simulation laboratory
		Collaboration with research centers, technology and innovation centers, evidence-based practice centers, quality improvement organizations, international competency-based training programs etc.
**Governance**	**Regulation and monitoring:** the function of governance in an organization is to set aims and to monitor and regulate the movement of the organization towards the attainment of these aims	Assessment program for faculty and trainees

**Table 2:** competency-structured presentation and discussion: applying the CanMEDS roles in bronchial asthma

Competency	Activities
**Medical expert**	Hypothesis-driven history taking and physical examination
	Patient- and family-centered history-taking and management decisions e.g. use of decision-aids
	Essential technical/procedural skills (pulse oximetry, peak flow meter recording, use of inhaler devices etc.)
	Essential investigations/imaging (choosing wisely)
	Use of calculators and scoring tools
	Emergency medical interventions
	Monitoring response to treatment
	Discharge planning/criteria for discharge
**Communicator**	Presentation skills feedback
	Counseling skills/ breaking bad news
	Motivational and therapeutic communication
**Collaborator**	Essential consultations and referrals e.g. pulmonology, pulmonary educator, allergologist etc.
**Advocate**	Essential educational input regarding asthma and its treatment, self-management plans etc.
	Risk factors counseling e.g. smoking, allergens
	Preventative and screening interventions e.g. vaccinations, bone mineral density assessment etc.
	Referral to patients' friends societies & support groups
**Leader**	Interventions to reduce cost of care/length of stay
	Quality indicators/audit of asthma care
	Economic comparisons of various interventions
**Scholar**	Evidence-based resources for asthma guidelines, protocols
	Asthma societies websites
	Update on new studies on asthma
**Professional**	Ethical challenges in asthma e.g. intubation or not, unorthodox treatments, refusing steroid therapy etc.

## Conclusion

Systems Thinking is a well-tried, evidence-based concept for creative system redesign that is advocated for social systems transformational change including medical education. Its implementation tools are extremely useful in guiding the leaders in medical education in realigning their curricula with the agreed-upon and desired educational and social outcomes. Reaching these crucial outcomes needs more than just “putting more pressure on the gas pedal”. It requires “a shifting of gears”!
